# Dietary Geranylgeraniol Mitigates Pain-Associated Behaviors via Improving Mitochondrial Function and Colon Integrity and Suppressing Neuroinflammation in Male Diabetic Neuropathy Rats

**DOI:** 10.3390/ijms262412133

**Published:** 2025-12-17

**Authors:** Chwan-Li Shen, Xiaobo Liu, Jay J. Cao, Volker Neugebauer, Jonathan M. Miranda, Moamen M. Elmassry, Dale M. Dunn, Jannette M. Dufour

**Affiliations:** 1 Department of Pathology, Texas Tech University Health Sciences Center, Lubbock, TX 79430, USA; xiaobo.liu@ttuhsc.edu (X.L.); dale.dunn@ttuhsc.edu (D.M.D.); 2 Center of Excellence for Integrative Health, Texas Tech University Health Sciences Center, Lubbock, TX 79430, USAjannette.dufour@ttuhsc.edu (J.M.D.); 3 Center of Excellence for Translational Neuroscience and Therapeutics, Texas Tech University Health Sciences Center, Lubbock, TX 79430, USA; 4 Grand Forks Human Nutrition Research Center, U.S. Department of Agriculture-Agricultureal Research Service (USDA-ARS), Grand Forks, ND 58202, USA; jay.cao@usda.gov; 5 Department of Pharmacology and Neuroscience, Texas Tech University Health Sciences Center, Lubbock, TX 79430, USA; 6 Garrison Institute on Aging, Texas Tech University Health Sciences Center, Lubbock, TX 79430, USA; 7 Department of Cell Biology and Biochemistry, Texas Tech University Health Sciences Center, Lubbock, TX 79430, USA; jonathan.m.miranda@ttuhsc.edu; 8 Department of Molecular Biology, Princeton University, Princeton, NJ 08540, USA; elmassry@princeton.edu; 9 Department of Medical Education, Texas Tech University Health Sciences Center, Lubbock, TX 79430, USA

**Keywords:** geranylgeraniol, diabetic neuropathy, behaviors, gut microbiome, mitochondria homeostasis, brain, rats

## Abstract

Growing evidence highlights the links between diabetic neuropathy (DNP), gut dysbiosis, mitochondrial dysfunction and neuroinflammation in colon and bone microstructure deterioration. Geranylgeraniol (GG) shows neuroprotective and osteoprotective capacity. Our study examines GG’s effects on pain-associated behaviors, glucose homeostasis, gut microbiota, mitochondrial homeostasis, and bone microstructure in DNP rats. We randomly assigned 27 male Sprague Dawley rats to three groups (*n* = 8–10/group): a control group (regular low-fat diet), a DNP group (high-fat diet + a single dose of 35 mg/kg streptozotocin), and a GG-treated DNP group (a single dose of 35 mg/kg streptozotocin + GG at 800 mg/kg in diet) for 6 weeks. Nocifensive response was assessed via the von Frey test and an open field test, and the elevated plus maze was used to assess anxio-depressive behaviors. The mRNA expression levels of tight junction protein, mitochondrial homeostasis, and neuroinflammation were measured in the colon using qRT-PCR. We collected fecal samples for microbiota composition analysis with 16S rRNA gene sequencing and analyzed by QIIME 2. All other data were analyzed via one-way ANOVA followed by post hoc Tukey’s multiple comparison. *p* < 0.05 was defined as statistical significance. Our study showed GG’s ability to mitigate mechanical hypersensitivity and anxio-depressive behavior in rats with DNP. GG supplementation did not improve glucose homeostasis (i.e., glucose intolerance, insulin sensitivity, pancreatic β-cell dysfunction) and bone microstructure. GG increased alpha-diversity without changing microbial abundance. DNP rats exhibited elevated Clostridium *sensu stricto* and reduced Eubacterium *coprostanoligenes*, *Lachnospiraceae*, *Oscillospiraceae*, and *Peptococcaceae* compared with controls. GG did not reverse DNP-induced gut dysbiosis but increased colonic claudin-3 (tight junction), MFN1 (mitochondria fusion), and TFAM (mitochondria biogenesis), while reducing FIS1 (mitochondria fission), GFAP (glial activation), P62 and PINK1 (mitophagy), and TNFα (inflammation). Functionally, GG reduced pain behaviors, improved intestinal integrity and mitochondrial homeostasis, increased alpha-diversity, and suppressed neuroinflammation, but did not improve glucose homeostasis or bone microstructure in obese DNP rats.

## 1. Introduction

The growing number of individuals with type 2 diabetes mellitus (T2DM, the major form of diabetes in 90–95% of all diabetes cases) is a major global health issue [1]. Between 7% and 75% of adults with diabetes have neuropathic pain (DNP) [2].

The primary causes of T2DM include glucose intolerance, insulin resistance, and pancreatic β-cells dysfunction, all of which correlate with chronic inflammation, oxidative stress, lipotoxicity, and endoplasmic reticulum stress [3,4]. Mitochondrial dysfunction’s role in colon health and gut microbiota dysbiosis is an emerging area of research in T2DM progression, based on the proposed microbiota–gut–brain axis [5,6,7]. Mitochondrial dysfunction in the colon may be partly responsible for the pathogenesis of T2DM by causing insulin resistance, impaired glucose metabolism, pancreatic β-cells dysfunction, and excessive oxidative stress [8]. Disruption of oxidative phosphorylation, which leads to decreased ATP production and imbalanced energy metabolism within the colon, can alter the normal functioning of colon nerves and lead to GI symptoms associated with DNP [9]. Gut microbiome imbalance (dysbiosis) can increase intestinal (colonic) permeability, resulting in the release of bacterial products that trigger an inflammatory response and further disrupt mitochondrial functioning. Dysbiosis creates a local environment of chronic inflammation and excessive oxidative stress, exacerbating colonic nerve damage and contributing to insulin resistance and impaired glucose homeostasis, further worsening DNP [8].

Diabetic neuropathy (DN), particularly diabetic peripheral neuropathy (DPN), is a widespread complication of T2DM. Approximately 30% of individuals with diabetes develop DN, with even higher prevalence reported when more sensitive diagnostic methods are used [10]. DPN typically begins with symmetrical numbness and sensory loss in the distal limbs and progresses to neuropathic pain in about 20% of patients [11,12]. However, its pathogenesis remains poorly understood because the condition is multifactorial. Disruption of Schwann cell homeostasis contributes to demyelination and subsequent neurodegeneration, which impairs sensory function. The dorsal root ganglia are especially vulnerable due to their limited myelination [13,14]. In addition, T2DM alters metabolic processes at the molecular level, activating multiple parallel pathways that collectively promote neural injury [10].

Research shown that DN can significantly affect bone microstructure (both trabecular and cortical bone), leading to increased risk of bone fracture. In trabecular bones, diabetic neuropathy can cause reduced bone mass and changed microarchitecture, including decreased trabecular number, thickness, and connectivity, as well as increased trabecular separation [10]. DN can also cause reduced cortical thickness and density, making cortical bone more susceptible to fractures [15].

Treatment options for DN or DNP are scarce and mainly palliative, such as alpha-lipoic acid and benfotiamine, which are dietary supplements with limited clinical evidence in DNP [16]. Given that dietary intervention is a first-line approach for DNP management, there is a compelling need for new strategies targeting both metabolic and neuropathic aspects of the condition.

In recent years, dietary bioactive compounds have gained attention for their potential to modulate complex disease pathways, particularly those involving chronic inflammation, mitochondrial dysfunction, and impaired glucose metabolism. Geranylgeraniol (GG or GGOH) is an isoprenoid found in grains, vegetables, and fruits, and has shown potential health benefits in glucose homeostasis [17], lipid metabolism, bone health [18,19] and neuropathic pain alleviation [20] in animal studies. Although direct clinical evidence for GG in T2DM is limited, its biological activities suggest potential therapeutic relevance. GG is a precursor to geranylgeranyl pyrophosphate (GGPP), which regulates glucose intake in skeletal muscle by influencing insulin signaling and promoting GLUT4 translocation [18,21,22,23]. Depletion of GGPP (e.g., by statin therapy) has been shown to impair glucose uptake, suggesting that GG supplementation may restore insulin signaling in this context [23]. GG also exhibits anti-inflammatory properties by suppressing NF-κB signaling and modulating the NLRP3 inflammasome in macrophages and microglial cells, both of which play a role in causing insulin resistance and neuropathic pain [24,25,26]. Furthermore, GG has been reported to preserve mitochondrial function in skeletal muscle and bone cells under diabetic or bisphosphonate-challenged conditions, which may be relevant to T2DM-associated sarcopenia and skeletal fragility [27,28]. The findings of these studies hint that GG can help regulate several T2DM-related pathologies, including glucose intolerance, inflammation, gut dysbiosis, bone deterioration, and chronic pain.

A high-fat-diet (HFD) plus a single low dose of streptozotocin (STZ) is a commonly used and well-established T2DM rat model that produces clinical symptoms linked to DNP [29,30,31]. We previously reported that GG supplementation improved glucose tolerance, insulin sensitivity, and bone microstructure in obese (HFD) mice, likely by inhibiting pro-inflammatory factors and modulating gut microbiome composition [18]. However, it remains to be determined if GG supplementation can also modulate pain-associated behaviors and the microbiota-gut-CNS connection in DNP animals treated with HFD plus a single low dose of STZ. To address this knowledge gap, we tested the hypothesis that GG supplementation would reduce mechanosensitivity and anxio-depression-like behaviors in DNP rats and that such effects are the result of improved glucose homeostasis, gut microbiome composition, and mitochondrial homeostasis in the colon and bone microstructure.

## 2. Results

### 2.1. Behavioral Outcomes

**Figure 1:**
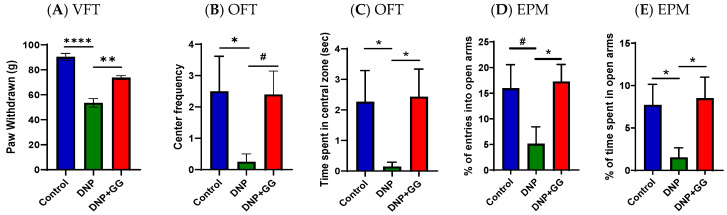
Effects of dietary geranylgeraniol supplementation on mechanical threshold as assessed by von Frey test (VFT) (**A**), anxio-depressive behaviors assessed as avoidance of open space in the open field test (OFT) (**B**,**C**) and of open-arms in the elevated plus maze (EPM) (**D**,**E**). Group assignment includes the control group (AIN-93G diet and single PBS injection), the DNP group (high-fat diet and single STZ injection), and the DNP + GG group (high-fat diet, single STZ injection, and geranylgeraniol at 800 mg/kg diet) for 6 weeks. Data is expressed as ±SEM. *n* = 9 per group and analyzed by one-way ANOVA followed by post hoc Tukey’s test. ^#^ 0.05 < *p* < 0.1, * *p* < 0.05, ** *p* < 0.01, **** *p* < 0.0001 for comparisons between control group and DNP group.

GG supplementation’s effects on behaviors associated with DNP were determined using the von Frey test (VFT) (Figure 1A), open field test (OFT) (Figure 1B,C), and elevated plus maze (EPM) (Figure 1D,E). At the end of the six-week study, (i) the DNP group exhibited much greater mechanosensitive pain compared to the control; (ii) compared to the vehicle-treated DNP group, mechanosensitive pain in rats was reduced by 35% (Figure 1A).

Regarding anxiety-like behaviors, the DNP group showed fewer entries (frequency) (Figure 1B) and spent less time in the open space (central areas) (Figure 1C) within the first 5 min compared to the control group, indicating anxiety-like avoidance behavior. Dietary GG supplementation (the DNP + GG group) significantly mitigated DNP-associated anxiety-like behaviors as reflected in increased entries (Figure 1B) and time spent (Figure 1C) in the center area. In the EPM, the DNP animals showed fewer entries (Figure 1D) and less time spent in the open arms (Figure 1E) than the control animals, while GG-supplemented animals (the DNP + GG group) showed significantly increased frequency (Figure 1D) and duration in the open arms (Figure 1E) during the first 5 min, indicating decreased aversion.

### 2.2. Glucose Homeostasis: Ipgtt and Ipitt

**Figure 2:**
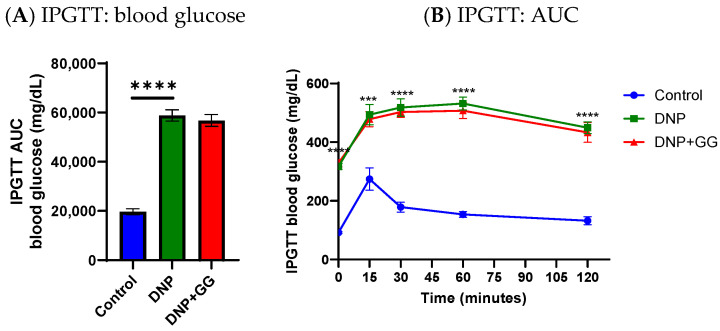

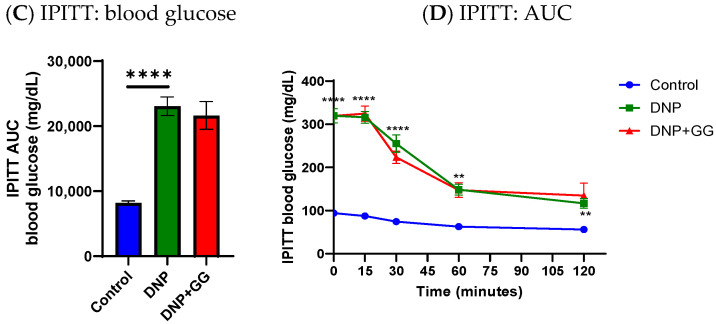
Effects of dietary geranylgeraniol supplementation on blood glucose tolerance and insulin resistance as assessed by IPGTT AUC (**A**), IPGTT (**B**), IPITT AUC (**C**), and IPITT (**D**). Group assignment includes the control group (high-fat diet group and single PBS injection), the DNP group (high-fat diet and single STZ injection), and the DNP + GG group (high-fat diet, single STZ injection, and geranylgeraniol at 800 mg/kg diet) for 6 weeks. Data are shown as mean ± SEM and analyzed by one-way ANOVA followed by post hoc Tukey’s test. *n* = 6–8 per group. ** *p* < 0.01, *** *p* < 0.001, **** *p* < 0.0001 for comparisons between control group and DNP group. Abbreviations: IPGTT, intraperitoneal glucose tolerance test; IPITT, intraperitoneal insulin tolerance test; AUC, area under the curve; DNP, diabetic neuropathic pain; DNP + GG, DNP plus 800 mg geranylgeraniol per kg in the diet.

After the 8-week feeding period, relative to the control group, the DNP group had significantly elevated glucose intolerance (Figure 2A,B) and insulin resistance (Figure 2C,D) as shown by blood glucose concentrations and AUC. Supplementation of GG into the diet (the DNP + GG group) for 6 weeks did not improve IPGTT and IPITT in the animals when compared to those without GG supplementation (the DNP group) (Figure 2A–D). These findings suggest that while GG proved beneficial in other physiological domains, its effects were insufficient to counteract the degree of glucose intolerance and insulin resistance already established in the DNP model over the 8-week period.

### 2.3. Insulin and Glucagon in the Pancreas

**Figure 3:**
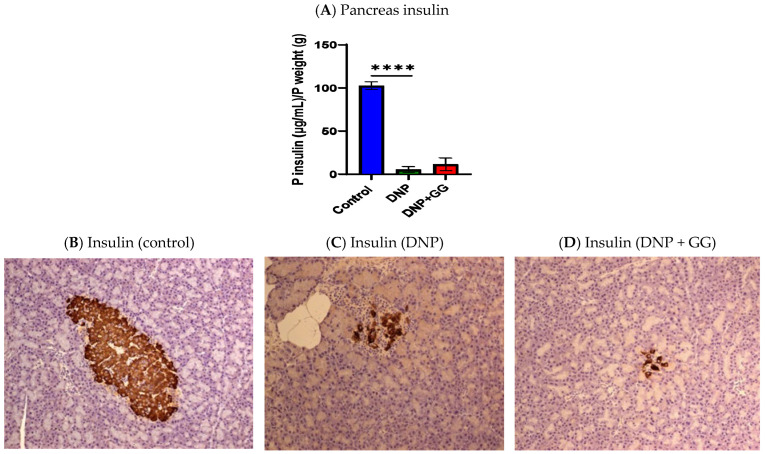

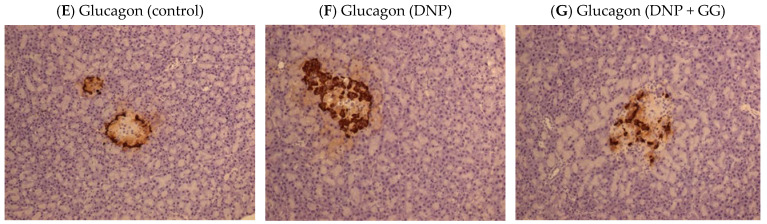
Effects of dietary geranylgeraniol supplementation on pancreas insulin concentration (**A**), pancreas insulin immunohistochemical analysis (**B**–**D**), and pancreas glucagon insulin immunohistochemical analysis (**E**–**G**). Group assignment includes the control group (AIN-93G diet and single PBS injection), the DNP group (high-fat diet and single STZ injection), and the DNP + GG group (high-fat diet, single STZ injection, and geranylgeraniol at 800 mg/kg diet) for 6 weeks. Data of insulin concentration is shown as mean ± SEM and analyzed by one-way ANOVA followed by post hoc Tukey’s test. *n* = 6–8 per group. **** *p* < 0.0001 for comparisons between control and DNP or DNP + GG. (**B**–**G**), magnification 20X.

At the study’s end, (i) pancreas insulin concentration in the DNP group was significantly reduced compared to the control group; and (ii) no difference in pancreas insulin concentration was found between the DNP group and DNP + GG group (Figure 3A).

Immunohistochemical staining of the pancreatic islets in the control group found normal islets with numerous insulin-positive beta cells (Figure 3B) and alpha cells arranged along the periphery of the islets (Figure 3E). Consistent with the decrease in pancreatic insulin content, the DNP group and the DNP + GG group both exhibited a loss of insulin-expressing cells (Figure 3F,G). Glucagon-expressing alpha cells were distributed throughout the islet in both the DNP group and the DNP + GG group (Figure 3C,D).

### 2.4. Gut Microbiota Analysis

**Figure 4:**
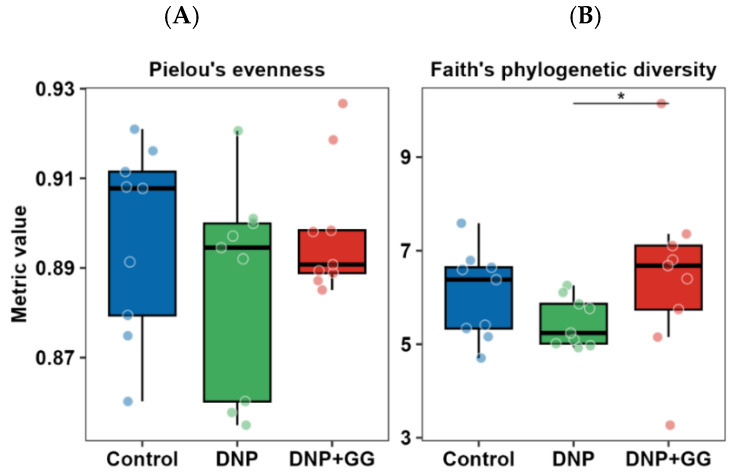
Effect of geranylgeraniol supplementation on the community evenness (**A**) and phylogenetic diversity (**B**) of the microbiome in cecal content. Group assignment includes the control group (high-fat diet group and single PBS injection), the DNP group (high-fat diet and single STZ injection), and the DNP + GG group (high-fat diet, single STZ injection, and geranylgeraniol at 800 mg/kg diet) for 6 weeks. *n* = 9 per group. * *p* < 0.05. Significance was determined using a Kruskal–Wallis test followed by Dunn’s test.

No significant differences were observed in the microbiome evenness (using Pielou’s evenness metric) among the three groups (Figure 4A). There was an increase in the diversity of the microbiome community (using Faith’s phylogenetic diversity metric) in the DNP + GG group vs. the DNP group (*p* < 0.05) (Figure 4B).

**Figure 5:**
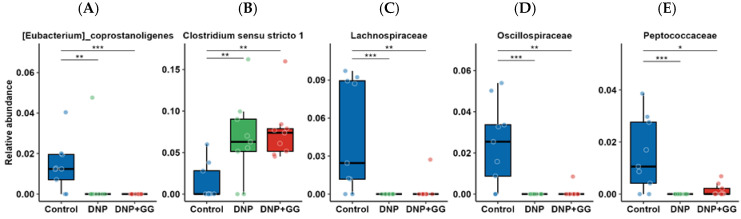
Effect of geranylgeraniol supplementation on the relative abundance of microbiome composition in the cecal content: (**A**) [Eubacterium]_coprostanoligenes, (**B**) *Clostridium sensu stricto 1*, (**C**) Lachnosporaceae, (**D**) Oscillospiraceae, (**E**) Peptococcaceae. Group assignment includes the control group (AIN-93G diet and single PBS injection), the DNP group (high-fat diet and single STZ injection), and the DNP + GG group (high-fat diet, single STZ injection, and geranylgeraniol at 800 mg/kg diet) for 6 weeks. *n* = 9 per group. *n* = 9 per group. * *p* < 0.05, ** *p* < 0.01, *** *p* < 0.001. Significance was determined using a Kruskal–Wallis test followed by FDR correction, then Dunn’s test.

Next, we focused on changes in the microbiome composition. We observed a shift in the relative abundance of some microbiome ASVs between the control and DNP groups. When compared to the control group, the relative abundance of *Eubacterium coprostanoligenes*, Lachnospiraceae, Oscillospiraceae, and Peptococcaceae (Figure 5A–E) decreased in the DNP group, while the relative abundance of *Clostridium sensu stricto 1* increased in the DNP group. These changes did not differ strongly from those in the DNP + GG group vs. the control group (Figure 5A–E). We observed no differences between the DNP + GG group and the DNP group.

### 2.5. mRNA Expression of Tight Junction Marker in Colon

**Figure 6:**
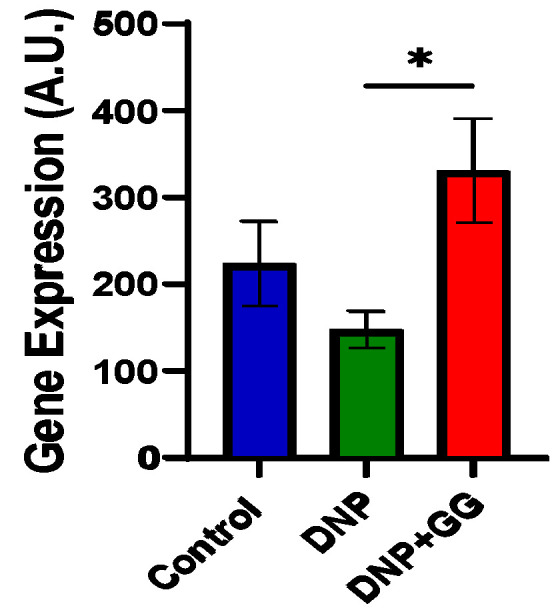
Effect of dietary geranylgeraniol supplementation on mRNA expression of claudin-3 in the colon. Group assignment includes the control group (AIN-93G diet and single PBS injection), the DNP group (high-fat diet and single STZ injection), and the DNP + GG group (high-fat diet, single STZ injection, and geranylgeraniol at 800 mg/kg diet) for 6 weeks. Data is expressed as mean ± SEM. *n* = 6–8 per group. Data was analyzed by one-way ANOVA followed by a post hoc Tukey’s test. * *p* < 0.05.

Figure 6 illustrates GG’s effects on mRNA expression of claudin 3, a tight junction maker in the colon of rats. The DNP group had lower claudin 3 mRNA expression levels than the control. However, after 8-week GG supplementation, the DNP + GG group had significantly enhanced claudin 3 mRNA expression levels when compared to both the DNP and control groups.

### 2.6. mRNA Expression of Mitochondrial Fusion, Fission, and Biogenesis Markers in Colon

**Figure 7:**
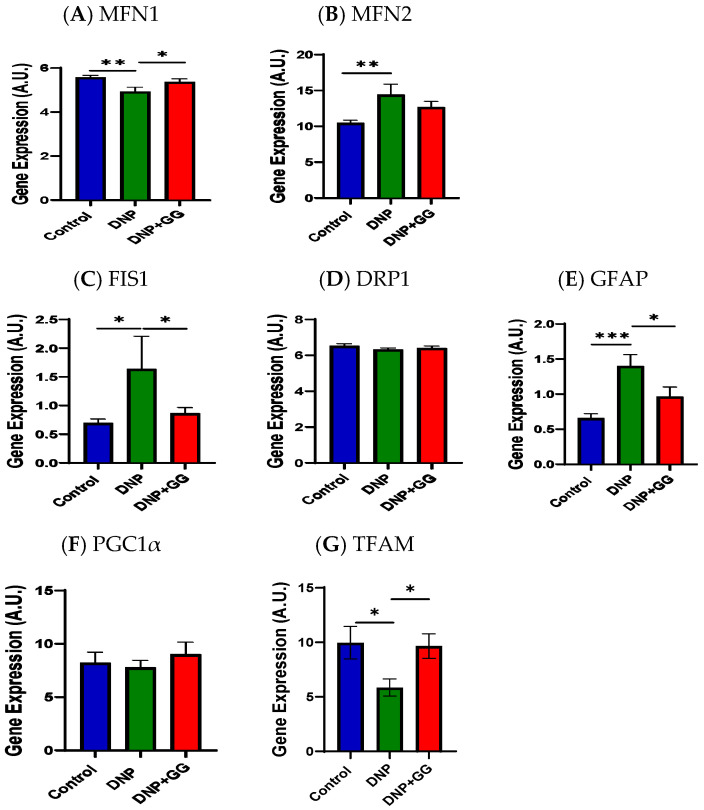
Effect of dietary geranylgeraniol supplementation on mRNA expression of MFN1 (**A**), MFN2 (**B**), FIS1 (**C**), DRP1 (**D**), GFAP (**E**), PGC1α (**F**), and TFAM (**G**) in the colon. Group assignment includes the control group (AIN-93G diet and single PBS injection), the DNP group (high-fat diet and single STZ injection), and the DNP + GG group (high-fat diet, single STZ injection, and geranylgeraniol at 800 mg/kg diet) for 6 weeks. Data is expressed as mean ±SEM. *n* = 6–8 per group. Data was analyzed by one-way ANOVA followed by a post hoc Tukey’s test. * *p* < 0.05. ** *p* < 0.01. *** *p* < 0.001. Abbreviations: MFN1, mitofusin 1; MFN2, mitofusin 2; FIS1, fission mitochondrial 1; DRP1, dynamin-related protein 1; GFAP, glial fibrillary acidic protein; PGC1α, peroxisome proliferative activated receptor alpha; TFAM, mitochondrial transcription factor A.

We examined the effects of GG supplementation on mitofusin 1 (MFN1) and mitofusin 2 (MFN2) mRNA expression for mitochondrial fusion in the colon. The DNP group had lower MFN1 gene expression levels than the control (Figure 7A) and greater MFN2 gene expression levels (Figure 7B). The GG administration resulted in increased MFN1 gene expression in the HSF + STZ-treated male rats (Figure 7A).

Figure 7C–E illustrate the fission-associated markers, namely the mitochondrial 1 (FIS1), dynamin-related protein 1 (DRP1), and glial fibrillary acidic protein (GFAP) mRNA expression in the colon of rats. FIS1 mRNA expression levels were higher in the colon of the DNP group than those in the control group, while the DNP + GG group had decreased FIS1 mRNA expression levels in the colon compared to those in the DNP group alone (Figure 7C). No differences were detected in DRP1 mRNA expression levels among all groups (Figure 7D). The DNP group exhibited higher GFAP mRNA expression levels than the control group, while GG supplementation (the DNP + GG group) suppressed such HFD + STZ-induced increased GFAP mRNA expression levels (Figure 7E).

We also examined the markers of mitochondrial biogenesis, namely peroxisome proliferative-activated receptor alpha (PGC1α) and mitochondrial transcription factor A (TFAM), in the colon. No significant differences were detected in PGC1α among all three groups (Figure 7F). Relative to the control group, the DNP group had lower TFAM mRNA expression levels in the colon. GG supplementation resulted in the colons of T2DM rats having greater TFAM mRNA expression levels (Figure 7G).

### 2.7. mRNA Expression of Mitophagy Markers in Colon

**Figure 8:**
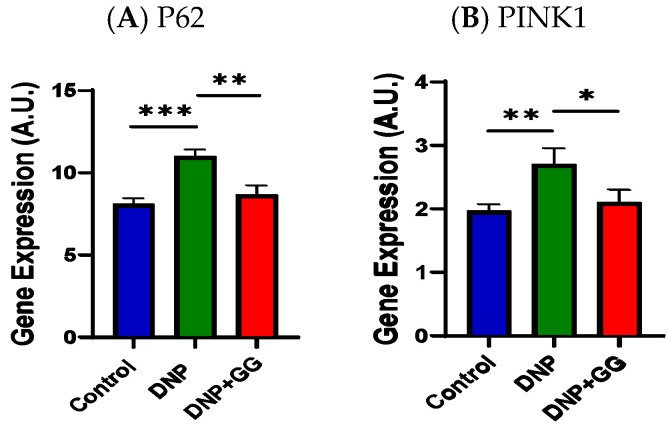
Effect of geranylgeraniol supplementation on mRNA expression of P62 (**A**) and PINK1 (**B**) in the colon. Data is expressed as mean ± SEM. *n* = 6–8 per group. Group assignment includes the control group (AIN-93G diet and single PBS injection), the DNP group (high-fat diet and single STZ injection), and the DNP + GG group (high-fat diet, single STZ injection, and geranylgeraniol at 800 mg/kg diet) for 6 weeks. Data was analyzed by one-way ANOVA followed by a post hoc Tukey’s test. * *p* < 0.05, ** *p* < 0.01, *** *p* < 0.001. Abbreviation: PINK1, PTEM-induced kinase 1.

We looked at GG supplementation’s effects on P62 (an autophagy degradation marker) and PTEM-induced kinase 1 (PINK1, initiation of the mitophagy process) in the colon. Compared to the control group, the DNP group had greater P62 and PINK1 mRNA expression levels in the animals’ colons. GG supplementation led to decreased P62 and PINK1 mRNA expression levels in the colon (Figure 8A,B).

### 2.8. mRNA Expression of Inflammation and Oxidative Stress Markers in Colon

**Figure 9:**
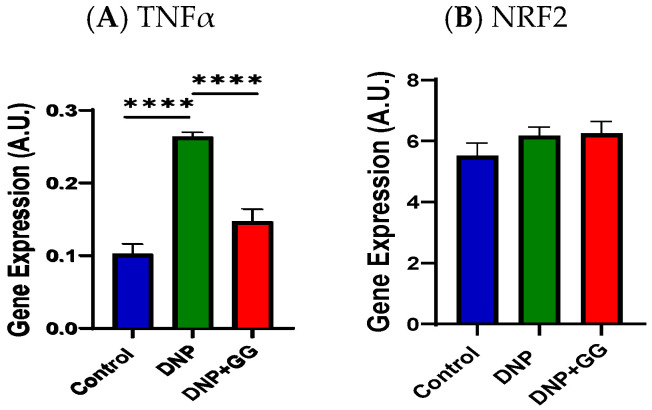
Effect of geranylgeraniol supplementation on mRNA expression of TNFα (**A**) and NRF2 (**B**) in the colon. Group assignment includes the control group (AIN-93G diet and single PBS injection), the DNP group (high-fat diet and single STZ injection), and the DNP + GG group (high-fat diet, single STZ injection, and geranylgeraniol at 800 mg/kg diet) for 6 weeks. Data is expressed as mean ± SEM. *n* = 6–8 per group. Data was analyzed by one-way ANOVA followed by a post hoc Tukey’s test. **** *p* < 0.0001. Abbreviations: TNFα, tumor necrosis factor α; NRF2, nuclear factor erythroid 2-related factor 2.

Figure 9 shows the effects of GG supplementation on tumor necrosis factor α (TNFα, inflammation marker) and nuclear factor erythroid 2-related factor 2 (NRF2, anti-oxidative stress marker) in the colon. As expected, the DNP exhibited increased TNFα mRNA expression levels in the colon relative to the control group. The GG-supplemented animals (the DNP + GG group) had decreased TNFα mRNA expression when compared to non-GG-supplemented animals (the DNP group) (Figure 9A). No significant difference was detected in NRF2 mRNA expression levels for all three groups (Figure 9B).

### 2.9. Bone Microstructure

Table 1 contains GG supplementation’s effects on the trabecular microstructure of the tibia. The DNP group exhibited significantly lower trabecular bone volume/total volume (BV/TV, %), trabecular number (Tb.N, mm^−1^), trabecular thickness (Tb.Th, mm), and connectivity density (Conn.Dn, mm^−3^), while increasing trabecular separation (Tb.Sp, mm) and structural model index (SMI) compared to the control. Supplementation of GG into the diet (the DNP + GG group) did not mitigate the HFD + STZ-induced microarchitectural deterioration, namely, BV/TV, Tb.N, Tb.Th, Tb.Sp, Conn.Dn, and SMI. In terms of cortical bone, the DNP group had decreased values of cross-sectional total area (T.Ar), cross-sectional bone area (B.Ar) and cortical thickness (Ct.Th) at the femoral cortical bone. GG supplementation did not restore the HFD + STZ-induced changes in T.Ar, B.Ar, and Ct.Th, resulting in the following order: control group > DNP group = DNP + GG group.

Data are expressed as mean ± SEM, *n* = 9. Group assignment includes the control group (high-fat diet group and single PBS injection), the DNP group (high-fat diet and single STZ injection), and the DNP + GG group (high-fat diet, single STZ injection, and geranylgeraniol at 800 mg/kg diet) for 6 weeks. Mean values within a row without common superscript letters differ significantly among the three dietary treatments by one-way ANOVA followed by post hoc Tukey’s test at *p* < 0.05. Abbreviations: B.Ar, cross-sectional bone area; B.Pm, bone perimeter; BV, bone volume; BV/TV, bone volume/total volume; Conn.Dn, connectivity density; Ct.Th, cortical thickness; Me.Ar, cross-sectional medullary area; T.Ar, cross-sectional total area; Tb.N, trabecular number; Tb.Sp, trabecular separation; Tb.Th, trabecular thickness; TV, total volume.

## 3. Discussion

This study highlights dietary GG’s beneficial effects in mitigating pain-associated behaviors in a high-fat diet plus STZ rat model of DNP by improved intestinal integrity, favored mitochondrial homeostasis, increased gut microbiome alpha-diversity, and suppressed neuroinflammation in DNP animals without affecting glucose homeostasis or bone microstructure. These improved pain-associated outcomes may involve regulating the microbiota-colon-brain connection.

The novel findings of this study show that dietary GG supplementation attenuates DNP-induced mechanosensitivity as assessed by VFT (Figure 1A). GG’s anti-nociceptive effects in rats with NP are consistent with published studies showing that GG (extracted from Pterodon pubescens Benth seeds) has notable anti-allodynic properties during the acute phase of pain in a variety of pain models, in part via 5-HT_3_ serotonin receptor involvement [32,33]. In addition, GG may mitigate pain by suppressing the nuclear factor-κB (NFκB) signaling pathway [25,27] or promoting tissue repair and regeneration [34].

GG is a key component of the mevalonate pathway, which is essential for brain function. Maintaining adequate levels of GG improves processes like hippocampal long-term potentiation by increasing hippocampal levels of serotonin, a neurotransmitter linked to mood regulation [35]. GG-derived geranylgeranylacetone has been shown to mitigate depression-associated behaviors in the forced swim and tail suspension tests in a chronic mild stress mouse model of depression via the suppression of monoamine oxidase-A expression [35]. These findings suggest the anxiolytic-like effects of GG supplementation improved avoidance behavior in both the OFT and EPM (Figure 1B–E). Importantly, our results suggest that GG may exert neurobehavioral benefits not only through monoaminergic pathways but also via its broader roles in mitochondrial homeostasis and neuroinflammation, mechanisms that have been increasingly recognized in mood and anxiety regulation. Further studies integrating these pathways may help clarify how GG contributes to the observed behavioral improvements.

Recent studies have suggested a strong connection between gut dysbiosis and the progression of diabetic neuropathic pain (DNP), particularly through impaired intestinal barrier function and subsequent systemic inflammation due to translocated bacterial products like lipopolysaccharides [36,37,38]. Certain gut microbes, such as *Eubacterium coprostanoligenes* and *Oscillospiraceae*, support mucin secretion and maintain the intestinal mucus barrier, thereby limiting inflammation and microbial invasion [39,40,41]. Reduced abundance of these taxa has been associated with increased gastrointestinal or neuropathic pain [42,43]. In our study, DNP rats exhibited lower levels of *E. coprostanoligenes* and *Oscillospiraceae* (Figure 5), reduced intestinal integrity (Figure 6), and increased mechanosensitivity (Figure 1A), supporting the protective role of these taxa in mucosal health and pain modulation.

*Clostridium sensu stricto 1* has been linked to chronic pelvic pain and heightened immune activation [44,45] in DNP rats, suggesting that its pro-inflammatory potential may be generalizable across pain conditions. Although the specific contribution of *Clostridium sensu stricto 1* to diabetic neuropathy remains to be determined, accumulating evidence implicates mucosal barrier disruption [46,47] and peripheral inflammatory signaling [46,48], both of which can sensitize nociceptive pathways. Moreover, decreased abundance of *Lachnospiraceae*, a butyrate-producing family implicated in gut-brain axis communication and anti-inflammatory signaling [44,45], has been observed in DNP animals and may contribute to both nociceptive and mood-related symptoms (Figure 1A–E and Figure 5). Altered *Lachnospiraceae* abundance has also been observed in individuals with migraine and ME/CFS, likewise suggesting a contribution to a broader range of disease conditions [49,50]. These microbial shifts collectively indicate the involvement of a microbiota–gut–pain axis in DNP pathophysiology.

We found that GG supplementation promoted claudin 3 mRNA expression in the colon (Figure 6), suggesting that GG enhanced colonic integrity in the rats with DNP. This positive influence on gut integrity is consistent with previous reports that GG supplementation might have prebiotic potential to promote gut microbiota growth and benefit gut health in obese mice by modifying the gut microbiome’s composition and suppressing inflammation [18]. While Chung’s study reported increased relative abundance of *B. pullicaecorum* and decreases in *D. longicatena* following GG supplementation [18], we observed that GG supplementation increased alpha diversity without reverting DNP-caused dysbiosis. The discrepancies in the gut microbiome profiles by GG may be attributed to several factors: (i) the use of different animal models (HFD-induced obese T2DM mouse model in Chung’s study vs. HFD + STZ-induced rat DNP model in this study), (ii) differing durations of GG intervention (14 weeks vs. 6 weeks), and (iii) differential effects on T2DM progression, as indicated by IPGTT and IPITT (improved glucose tolerance and insulin sensitivity in Chung’s study [18] vs. no change in glucose tolerance and insulin resistance in this study).

Somewhat surprisingly and contrary to our hypothesis, GG supplementation did not improve the HFD + STZ-induced negative impacts on glucose homeostasis (i.e., glucose intolerance, insulin resistance, low pancreatic insulin expression) and bone microstructure of trabecular and cortical bones. This is likely due to STZ-induced diabetes, which is known to cause more severe damage to beta cells in the pancreas and more profound metabolic disturbances than diet-induced models [51]. However, the HFD + STZ model is commonly used to study both early- and late-stage diabetic neuropathy. STZ-induced β-cell damage leads to sustained insulin deficiency, hyperglycemia, and downstream dysregulation that are difficult to reverse through dietary interventions. Moreover, reduced insulin levels may lead to increased glucagon secretion, further aggravating hyperglycemia through enhanced hepatic glucose output. While we did not measure glucagon in this study, this mechanism aligns with the observed metabolic profile of DNP rats and may contribute to the lack of improvement with GG. Given that GG is a dietary compound rather than a pharmacological agent, its modulatory effects may not be potent enough to counteract the rapid and severe metabolic disruptions caused by STZ within a relatively short intervention period.

In addition, GG supplementation had no affect NRF2 mRNA expression in the colon. NRF2 is a key regulator of antioxidant defense and metabolic homeostasis, and its activation has been shown to protect pancreatic β-cells from oxidative stress while enhancing insulin sensitivity [52]. If GG also failed to activate NRF2 in the pancreas, this could further explain its limited effects on glucose regulation and pancreatic hormone expression in this model.

In this study, mitochondrial homeostasis refers to the dynamic equilibrium between markers of mitochondrial fusion (such as MFN1, MFN2), fission (such as FIS1, DRP1), biogenesis (such as PGC1α, TFAM), and autophagy (such as PINK1) [53]. The interplay between imbalanced mitochondrial homeostasis and nerve cell damage is closely linked to DNP [54,55]. Mitochondrial dysfunction, especially impaired ATP energy production and increased oxidative stress/inflammation [37,56], is partly responsible for the pathophysiology of pain [57] and depression [58,59,60]. MFN1 and MFN2 play a large part in glucose-stimulated insulin secretion (GSIS) by regulating mitochondrial DNA (mtDNA) content, suggesting the combined MFN1/2 function is critical [61,62,63]. This MFN1/2 regulation occurs through the control of TFAM expression, which is vital for mtDNA maintenance and glucose regulation in diabetes [64]. Thus, the altered expression of MFN1/2 in this study revealed GG’s ability to overcome the mitochondrial changes caused by DNP (Figure 7A,B). Both FIS1 and DPR1 are key proteins involved in mitochondrial fission [65,66]. In our study, GG reversed the DNP-induced increase in FIS1 expression (Figure 7C), while DRP1 remained unchanged (Figure 7D). These findings may relate to the timing of the GG intervention or sample collection. Nevertheless, the results indicate that mitochondrial fission dynamics were altered in DNP and that GG was able to counteract at least part of this disruption. In terms of mitochondrial biogenesis [67,68,69], we observed a disturbance in TFAM expression in this study (Figure 7G). This change was reversed by GG supplementation, suggesting that GG may help restore mitochondrial integrity. Both PINK1 (a mitochondrial kinase) and P62 (also known as sequestosome-1) are involved in the mitophagy process [70,71]. In the present study, levels of PINK1 and PG62 mRNA expression, which were increased in the DNP group, were reduced following GG supplementation, supporting the notion that GG helps stabilize mitochondrial homeostasis under neuropathic conditions. The beneficial influences of GG in mitochondrial homeostasis align with a previous report that GG supplementation mitigates soleus muscle atrophy via changes in mitochondrial quality in diabetic rats [27].

The present study strongly suggests a connection between colonic mitochondrial dysfunction and DNP progression, which corroborates previously reported links between mitochondrial dysfunction and inflammatory bowel disease [72]. Moreover, it offers a potential explanation for the mechanism by which GG alleviates T2DM-associated NP behaviors. These beneficial effects of GG on colonic mitochondrial homeostasis/quality in DNP rats are consistent with those in the soleus muscle of diabetic rats [27].

Besides glucose homeostasis, we also did not find improved bone microstructure due to GG supplementation in the high-fat-diet-induced obese rats with DNP. Previously, we demonstrated that GG supplementation mitigated bone microstructure deterioration in HFD-induced obese mice [18]. We speculate that such STZ-induced detrimental impacts on bone microstructure could counter the possible osteoprotective effects of GG supplementation in the DNP rats.

Our study has several limitations. First, due to the limited availability of colon tissue samples, we were unable to assess protein expression of key target genes to confirm the mRNA results. Second, we did not intend for our study to comprehensively investigate the molecular mechanisms of how GG influences diabetic neuropathic pain. Although some mechanisms were discussed, not all were covered. Third, although the use of the HFD + STZ model was necessary to induce DNP-like symptoms, its severity may have obscured potential improvements in glucose metabolism and related parameters. Fourth, the relatively short duration of GG supplementation may have curtailed its therapeutic impact. Future studies with more samples and extended treatment periods are warranted to validate these findings at the protein level (e.g., via Western blot or immunohistochemistry) and to further elucidate the mechanisms underlying GG’s effects.

We acknowledge that gabapentin, pregabalin, and duloxetine are widely endorsed as first-line treatments for painful diabetic neuropathy across major management guidelines issued by diabetes societies [73,74]. However, our decision not to use a pharmacological agent as a positive control in the present study was based on the exploratory and mechanistic scope of this investigation. Specifically, the primary objective of this work was to delineate GG’s influence on neuroimmune signaling, mitochondrial homeostasis, gut–barrier integrity, and nociceptive behavior. Several prior DPN studies [75,76,77] focused on molecular or mechanistic pathways have similarly omitted comparator agents when the goal was to identify biological signatures rather than benchmark relative analgesic magnitude. While including a positive control can strengthen translational interpretation, meaningful mechanistic insights can be obtained without it when the experimental focus centers on pathway-level modulation. Instead of a pharmacological control, our design prioritized careful nutritional modulation, behavioral evaluation, molecular readouts, and multi-level mechanistic endpoints (mitochondrial markers, neuroinflammation, gut integrity, and microbiome features), all of which provide meaningful internal validity. The present study lays the groundwork for future investigations to test GG complement standard DNP medications or metabolic modulators to assess potential additivity, synergy, or comparative efficacy under varied disease conditions.

In conclusion, this study provides the first evidence that 8-week dietary supplementation with geranylgeraniol (GG) alleviates pain-associated and anxio-depressive behaviors in a rat model of diabetic neuropathy, likely through improving colonic mitochondrial homeostasis, maintaining intestinal barrier integrity, and modulating gut microbiota. Although GG did not modify glucose homeostasis or bone microstructure under the severe HFD + STZ-induced diabetic condition, its beneficial effects on neural and gastrointestinal parameters identify GG as a potential candidate for targeting the microbiota–gut–brain axis in diabetic neuropathic pain. These findings provide the scientific premise for further mechanistic investigations, including the signaling pathways by which GG influences mitochondrial regulation and neuroimmune activation, as well as validation in additional disease models with varying severity and metabolic backgrounds. Ultimately, long-term intervention studies evaluating GG as a dietary supplement for managing complications of type 2 diabetes are necessary to show its translational value in the prevention or management of DNP. Testing GG alongside standard neuropathic pain medications or metabolic modulators is also needed to assess potential additivity, synergy, or comparative efficacy under various disease conditions.

## 4. Methods and Materials

### 4.1. Animals and Treatments

We received twenty-seven 5-week-old male Sprague Dawley (SD) rats from Envigo, Indianapolis, IN, USA. Animals were individually housed under a 12 h light–dark cycle with water and food ad libitum. Room temperature was controlled at 70 ± 2 °F, with a humidity of 30–50%. The Institutional Animal Care and Use Committee (IACUC number #19175) at Texas Tech University Health Science Center, Lubbock, TX, USA, approved our animal study protocol.

After one week of acclimation, we randomly assigned the animals by their body weights to 3 groups: the control group (*n* = 11), the DNP group (*n* = 11), and the DNP + GG group (*n* = 11). Animals in the control group were fed an AIN-93G diet (D10012G, Research Diet Inc., New Brunswick, NJ, USA) throughout the study period and received one i.p. injection of phosphate-buffered saline after 2 weeks of feeding. Animals in the DNP group were fed a high-fat diet (HFD) (D12492, Research Diet Inc., New Brunswick, NJ, USA) throughout the study period and after 2 weeks of feeding received one i.p. injection of streptozotocin (STZ, Sigma-Aldrich, Inc., St. Louis, MO, USA) at 35 mg/kg body weight to induce diabetes [51,78]. Animals in the GG group were fed HFD + 800 mg GG/kg diet throughout the study period, and after 2 weeks of feeding, received one i.p. injection of STZ. Non-fasting blood sugar was measured in all STZ-treated mice 10 days after the injection, and animals without hyperglycemia (>300 mg/dL) were excluded from the study as non-responders to STZ induction. After confirmation of hyperglycemia, there were 11 animals in the control group, 9 animals (2 exclusions) in the DNP group, and 10 (1 exclusion) animals in the GG group.

The dietary intervention lasted 8 weeks. The dose of 800 mg/kg/day for the GG group was selected based on previous studies showing its beneficial effect on glucose homeostasis and bone microstructure in obese mice [18]. The GG (85% purity) was gifted to us from American River LLC, Hadley, MA, USA. GG is classified as “Generally Recognized as Safe” (GRAS), and no safety concerns have been reported at the 800 mg/kg diet dose. We assessed body weight, food consumption, and water intake weekly.

We performed a power analysis based on preliminary data and previous studies from our laboratory [79]. To detect a significant change in pain hypersensitivity levels at α = 0.05 with 90% power, a sample size of 8–10 rats per group was sufficient. Thus, we used *n* = 11 rats per group to ensure adequate statistical power. After removing non-hyperglycemic animals (2 in the DNP group and 1 in the GG group) from our study, there was still enough statistical power (*n* = 9–11 per group) for the outcome assessment.

### 4.2. Behavioral Assessment

Prior to dietary feeding and STZ injection (baseline) and one week before sample collection (end of study), we assessed the behaviors of the animals as described below. The pain mechanosensitivity of the animals’ left paw was assessed using an Electronic von Frey Aesthesiometer (model: IITC Life Science, Woodland Hill, CA, USA) according to the published method [79]. Delayed withdrawal indicated decreased pain sensitivity, which is typically related to higher diabetic neuropathy levels in a high-fat diet plus a single dose of STZ animal model. We assessed the anxio-depressive behaviors of animals during the first 5 min of a 15 min exploratory time in the open field test (OFT), where duration and entries into the center area were recorded according to the protocol reported in our previous publication [80], and in the elevated plus maze (EPM), where the duration and entries into the open arm were recorded as reported in our previous publication [79]. The first 5 min were chosen for the analysis of anxio-depressive behaviors to capture the animal’s most robust and natural anxiety-related behavior when faced with a novel, aversive environment. Behavior after the first 5 min reflects a combination of habituation, learning, and other factors, potentially confounding the assessment of anxio-depressive behaviors.

### 4.3. Glucose Homeostasis and Pancreas Assessment

At baseline and one week before sample collection, we conducted an intraperitoneal glucose tolerance test (IPGTT) and an insulin tolerance test (IPITT) on animals fasted for four hours. Blood glucose levels were measured at 0, 15, 30, 60, and 120 min after intraperitoneal injection of glucose (2 g/kg body weight) or insulin (1 U/kg body weight). Both IPGTT and IPITT tests were performed between 8 am and 12 pm on the test dates. The different groups were tested in the same order for all tests (control group, then DNP group, and then GG group). This approach ensured consistency in timing across groups, minimized any variability introduced by the testing order, and allowed direct comparison of data between groups.

IPGTT and IPITT area under the curve (AUC) were calculated using the trapezoidal method. Total pancreas insulin content was measured using a mouse insulin ELISA (EMD Millipore Co., Billerica, MA, USA) after acetic acid extraction. The immunohistochemical staining for insulin and glucagon in the pancreatic tissue sections was performed using respective primary and secondary antibodies. The tissue sections were counterstained with Mayer’s hematoxylin based on our published work [81].

### 4.4. Sample Collection

Prior to sample collection, the animals were fasted for 4 h and blood was drawn from the heart of animals anesthetized using isoflurane. After centrifugation, the serum samples were collected and stored at −80 °C for later analysis. Pancreas tissues were harvested and either stored in acetic acid at −80 °C for insulin extraction or fixed in Z-fix (AnaTech Ltd., Battle Creek, MI, USA) at room temperature and embedded in paraffin for later histological measurement [82]. The colon and spinal cord were collected, immersed in liquid nitrogen, and kept at −80 °C for later mRNA analysis. Cecal feces were harvested, immersed in liquid nitrogen, and stored at −80 °C for later gut microbiome analysis. The right tibia and femur were harvested, cleaned of adhering soft tissues, and stored in 70% ethanol at 4 °C for bone microstructure analysis.

### 4.5. Gut Microbiota Profile

Fecal DNA was extracted using PowerFecal DNA isolation kit (Qiagen LLC, Germantown MD, USA). The amplicon sequencing of V4 variable region of the 16S rRNA gene was conducted at Molecular Research LP (Shallowater, TX, USA) as previously described [80]. Raw sequencing data was deposited under the access number PRJNA936631 in the National Center for Biotechnology Information BioProject database.

### 4.6. mRNA Expression Measurement

We extracted the total RNA of the spinal cord (lower region) and distal colon using the RNAzole RT (Molecular Research Center Inc., Cincinnati, OH, USA), quantified total RNA, reverse transcribed total RNA into cDNA, and amplified the targeted genes and β-actin (housekeeper gene) using the primers (Supplementary Table S1) for primer sets of their respective genes. We calculated gene expression using the formula 2 ^−(∆CT×1000)^ [83].

### 4.7. Bone Microstructure Assessment

We assessed the bone microstructure of proximal and cortical bones of the tibia and femur using micro-computed tomography (Scanco µCT 40; SCANCO Medical AG, Brüttisellen, Switzerland). We analyzed the parameters for trabecular bones in the tibia and femur and for cortical bone in the femur according to published works [80,81].

### 4.8. Statistical Analyses

We presented the results as mean ± standard error of the mean (SEM). Normality was evaluated by visual inspection of residual and Q–Q plots, consistent with recommended practices for small-sample animal studies, and ANOVA was applied only when data reasonably conformed to Gaussian distribution assumptions. We analyzed the GTT and ITT data at each measured time point, behaviors, bone microstructure, and mRNA expression by one-way ANOVA test followed by post hoc Tukey’s test using GraphPad Prism software (version 9.0, GraphPad Software, Boston, MA, USA). Microbiome 16S rRNA amplicon sequencing data were analyzed using QIIME 2 software based on previously published work [80]. *p* value < 0.05 indicates a significant difference for all results. ^#^ 0.05 < *p* < 0.01, * *p* < 0.05, ** *p* < 0.01, *** *p* < 0.001, **** *p* < 0.0001.

## 5. Conclusions

This study highlights the beneficial effects of dietary GG supplementation on the mitigation of diabetic pain-associated behaviors in a preclinical HFD + STZ mouse model through improving mitochondrial homeostasis and suppressing inflammation.

## Figures and Tables

**Table 1:** Effects of geranylgeraniol supplementation on bone microstructure properties of the tibia and femur in male rats with diabetic neuropathy.

Parameters	Control	DNP	DNP + GG	*p* Value
** Proximal tibia (trabecular bone) **				
BV/TV (%)	15.30 ± 0.93 ^a^	10.21 ± 1.14 ^b^	8.76 ± 0.90 ^b^	0.0044
Tb.N (mm^−1^)	3.02 ± 0.15 ^a^	2.16 ± 0.19 ^b^	1.89 ± 0.09 ^b^	<0.0001
Tb.Th (mm)	0.070 ± 0.001 ^a^	0.064 ± 0.002 ^b^	0.063 ± 0.002 ^b^	0.0029
Tb.Sp (mm)	0.33 ± 0.01 ^b^	0.51 ± 0.04 ^a^	0.56 ± 0.03 ^a^	<0.0001
Conn.Dn (mm^−3^)	55.85 ± 5.80 ^a^	38.37 ± 6.15 ^b^	30.39 ± 5.35 ^b^	0.0002
SMI	2.23 ± 0.06 ^b^	2.50 ± 0.07 ^a^	2.64 ± 0.08 ^a^	0.0009
** Femoral mid-dialysis (cortical bone) **				
T.Ar (mm^2^)	7.07 ± 0.19 ^a^	6.34 ± 0.16 ^b^	6.26 ± 0.22 ^b^	0.0099
B.Ar (mm^2^)	5.03 ± 0.09 ^a^	4.53 ± 0.10 ^b^	4.44 ± 0.13 ^b^	0.0010
Me.Ar (mm^2^)	2.04 ± 0.10	1.81 ± 0.07	1.82 ± 0.11	0.1719
B.Pm (mm)	17.09 ± 0.37 ^a^	16.07 ± 0.23 ^a^	16.06 ± 0.44 ^a,b^	0.0576
** Ct.Th (mm) **	** 0.619 ± 0.007 ^a^**	** 0.592 ± 0.010 ^b^**	** 0.578 ± 0.007 ^b^**	** 0.0002 **

Values sharing the same letter (a or b) are not significantly different; values with different letters indicate a statistically significant difference.

## Data Availability

The raw sequencing data (BioProject access number PRJNA936631) of 16S rRNA amplicon sequencing was deposited in the National Center for Biotechnology Information BioProject database. The original contributions presented in this study are included in the article. Further inquiries can be directed to the corresponding authors.

## Supplementary Materials

The following supporting information can be downloaded at: https://www.mdpi.com/article/10.3390/ijms262412133/s1.
