# Supplementary material for: Dietary Geranylgeraniol Mitigates Pain-Associated Behaviors via Improving Mitochondrial Function and Colon Integrity and Suppressing Neuroinflammation in Male Diabetic Neuropathy Rats
Source: Int J Mol Sci. 2025 Dec 17;26(24):12133. doi: 10.3390/ijms262412133 (PMC12733174; doi:10.3390/ijms262412133)
Supplement: Supplementary file 1 [file ijms-26-12133-s001.zip › ijms-3947864-supplementary.pdf]

Supplement Table S1. List of primers

Gene	Forward	Reverse
Claudin-3	5’-CCC AGC CTA CGG AGT TAC CC-3’	5’-TGC CGA TGA ATG CCG AAA CG-3’
MFN1	5’-AGC TCG CTG TCA TTG GGG AG-3’	5’-TCC CTC CAC ACT CAG GAA GC-3’
MFN2	5’-TCC TGA ACA ACC GCT GGG AT-3’	5’-GAT CCA CCA CGC CTA GCT CA -3’
FIS1	5’-CTG CGG TGC AGG ATG AAA GAC-3’	5’-GGC GTA TTC AAA CTG CGT GCT-3’
DRP1	5’-ACA ACA GGA GAA GAA AAT GGA GTT G-3’	5’-AGA TGG ATT GGC TCA GGG CT-3’
PGC1α	5’-CAG GAG CTG GAT GGC TTG GG-3’	5’-GGG CAA AGA GGC TGG TCC T-3’
TFAM	5’-GCT TCC AGG GGG CTA AGG ATG-3’	5’-TCG CCC AAC TTC AGC CAT TT-3’
P62	5’-CTG AGT CGG CTT CTG CTC CA-3’	5’-GCG GCT TCT CTT CCC TCC AT-3’
PINK1	5’-TCG GCC TGT CAG GAG ATC CA-3’	5’-CAT TGC AGC CCT TGC CGA TG-3’
GFAP	5’-AAT CTC ACA CAG GAC CTC GGC-3’	5’-AGC CAA GGT GGC TTC ATC CG-3’
TNFα	5’-GAA CTC CAG GCG GTG TCT GT-3’	5’-CTG AGT GTG AGG GTC TGG GC-3’
NRF2	5’- CTC TCT GGA GAC GGC CAT GAC T-3’	5’- CTG GGC TGG GGA CAG TGG TAG T-3’
B-actin	5’-ACA ACC TTC TTG CAG CTC CTC C-3’	5’-TGA CCC ATA CCC ACC ATC ACA-3’

Abbreviation:
DRP1, dynamin-related protein 1; FIS1, fission mitochondrial 1; GFAP, glial fibrillary acidic protein; MFN1, mitofusin 1; MFN2, mitofusin 2; NRF2, nuclear factor erythroid 2-related factor 2; PGC1α, peroxisome proliferative activated receptor alpha; PINK1, PTEN-induced kinase 1, TFAM, mitochondrial transcription factor A; TNF-α, tumor necrosis factor-α.
.
